# Knowledge of the abortion law and key legal issues of sexual and reproductive health and rights among recently arrived migrants in Sweden: a cross-sectional survey

**DOI:** 10.1186/s12889-023-15399-z

**Published:** 2023-03-23

**Authors:** Veronika Tirado, Anna Mia Ekström, Nicola Orsini, Claudia Hanson, Susanne Strömdahl

**Affiliations:** 1 Department of Global Public Health, Karolinska Institutet, Stockholm, Sweden; 2 Department of Infectious Diseases, Venhälsan, Södersjukhuset, Stockholm, Sweden; 3 London School of Hygiene and Tropical Medicine, London, UK; 4 Department of Medical Sciences, Infectious Medicine, Uppsala University, Uppsala, Sweden

**Keywords:** Sexual and reproductive health and rights, Abortion, Sexual consent, Knowledge, Rights, Laws, Survey, Migrants, Sweden

## Abstract

**Background:**

Sexual and reproductive health and rights (SRHR), including access and information on the laws and policies related to abortion, varies considerably between countries. Migrants may have limited knowledge of SRHR and related resources in their new country. This study investigates migrants’ knowledge of the right to safe and legal abortion and other associated factors including the recent law on sexual consent, the legal age for sexual consent and age to marry in Sweden.

**Methods:**

We conducted a cross-sectional study from 2018 to 2019 among recent migrants attending high schools or Swedish language schools. Descriptive statistics were computed on the knowledge of the Swedish abortion law and other legal aspects. Univariable and multivariable logistic regression analyses were conducted to assess if migrants’ socio-demographic characteristics were associated with knowledge (i.e. correct/incorrect) of the Swedish abortion law and other key SRHR-related legal issues.

**Results:**

Of the total 6,263 participants, 3,557 (57%) responded about whether it is legal to have an induced abortion in Sweden, and of these, 2,632 (74%) answered incorrectly. While more than half (61%) of the respondents knew the sexual consent law, nearly half (48%) did not know that sexual consent is also required for married couples. About 90% correctly responded that it is illegal to have sex with a minor (under the age of 15) and were aware of the legal age (18 years) to marry in Sweden. Incorrect knowledge of the Swedish abortion law was associated with being religious (adjusted odds ratio (AOR), 2.12; 95% confidence interval (CI), 1.42–3.15), not having previous sexual health education (AOR, 1.68; 95% CI, 1.38–2.05), coming from a country with predominantly restrictive abortion laws (AOR, 1.46; 95% CI, 1.16–1.84), low level of education (AOR, 1.29; 95% CI, 1.04–1.61) and having a temporary residence permit (AOR, 1.27; 95% CI, 1.02–1.57).

**Conclusion:**

We found a substantial lack of knowledge among migrants of reproductive age in Sweden regarding important laws and policies of SRHR, particularly the right to abortion. SRHR-related programmes and comprehensive sexual health education for recently arrived migrants could include components to increase knowledge of legal and safe abortions and other laws concerning SRHR.

**Supplementary Information:**

The online version contains supplementary material available at 10.1186/s12889-023-15399-z.

## Background

Evidence shows that in situations of conflict and forced migration, people’s sexual and reproductive health and rights (SRHR) are affected by limited access to contraceptives, unsafe sexual practices, unwanted pregnancies, unsafe abortions, and an increased risk of contracting and transmitting sexually transmitted infections (STIs), such as HIV [1, 2]. In addition to these risks, migration has also been associated with increased sexual abuse and gender-based violence (GBV) [3]. However, laws and policies related to SRHR vary widely across different countries and can violate, impose or support human rights and access to sexual and reproductive health services [4, 5]. Legislations surrounding SRHR consist of various domains, including abortion, which ranges from an uncomplicated health procedure to being prohibited altogether or allowed only upon request to save a woman’s life [6, 7]. Even in countries with liberal abortion laws, women often seek unsafe abortion methods and jeopardise their health in clandestine settings because of privacy issues and a lack of knowledge of abortion rights [8]. Previous research in several countries found that public knowledge about abortion laws is minimal [9–15]. Knowledge of abortion laws is important for migrants who often face challenges in the health system and language barriers and may lack information about their rights in a new setting [16].

### Migration and SRHR in Sweden

Over the past decade, Sweden has received a relatively larger number of refugees and other migrant groups than most other European countries, reaching an all-time high in 2015 with nearly 163,000 asylum seekers [17]. Recently arrived migrants or official family reunification migrants with a temporary residence authorisation and asylum seekers waiting for a decision from the Swedish Migration Agency often under-prioritise their SRHR. They may also encounter more significant legal uncertainties that hinder their awareness of sexual and reproductive health services [18]. Contraceptive counselling and abortion care are available for migrants without a residence permit, as well as for those with a permanent or temporary residence permit in Sweden [19]. To best respond to the various challenges and needs, inclusive integration policies for newly arrived migrants have been implemented in Sweden. For example, civic orientations and introduction programmes provide general information about the Swedish society and SRHR-related information [20]. However, only recently settled migrants with a residence permit based on the refuge or protection grounds can partake in the introduction programme, potentially excluding migrants, such as asylum seekers who are waiting for a residence permit and undocumented migrants, from receiving SRHR-related information in Sweden [21, 22].

### Key SRHR-related laws and policies in Sweden

Sweden has a long-standing history of progressive laws and policies related to the SRHR [23]. The Swedish Abortion Act was implemented in 1974, allowing pregnant women to request an induced abortion under gestational age limits regardless of reasons [24, 25]. Previous research in Sweden showed that migrant women are more likely to have an induced abortion than Swedish-born women because migrant women had less experience with and access to contraceptives and family planning [26]. Compulsory sexual health education programmes, including facts about sexuality, reproductive health, abortion and contraceptives, were introduced in all Swedish schools in 1955. The Swedish Association for Sexuality Education *Riksförbundet för sexuell upplysning* (RFSU) often provides these sexual health education programmes across Sweden [27]. However, religious traditions, beliefs, and resource limitations may hinder migrants’ access to age-appropriate sexual health education and information about SRHR [28]. For instance, a qualitative study found that migrant parents in Sweden who did not have previous sexual health education, experienced a clash of attitudes towards discussing sex and sexuality in their home countries and Sweden [29]. Norms and values are influenced by the person’s religious and cultural background, which may also affect the health-seeking behaviours in migrant communities [30].

Policy changes to SRHR have also occurred over the last five years in Sweden. As of 2018, the Swedish law on sexual consent changed the legal definition of how consent may be expressed verbally and non-verbally, thereby influencing what can be seen as illegal or rape during sexual encounters [31]. Previous research described that Swedish youth had contradictory interpretations of the new Swedish law on sexual consent. This study found that young people may consider a situation like ‘going home with someone after a night out’ as consent to sex (i.e. yes to sex) [32]. Non-consensual intercourse, even within marriage, has considerable human rights violations. In particular, young women may be exposed to GBV and loss of rights to their sexual integrity and control of contraceptive methods for family planning [31]. Other SRHR-related laws and policies in Sweden include the legal age (15 years) for consent to sexual activities and the legal age (18 years) to marry [33, 34]. Accurate information on laws and policies related to SRHR may prompt people, especially young migrants, to exercise their rights in all situations that the law applies. Lack of knowledge and not paying heed to the laws and policies surrounding SRHR can also lead to adverse effects, such as unexpected legal consequences and human rights violations [35].

### Rationale and aim

The World Health Organization (WHO) and the United Nations (UN) recognise that sexual and reproductive health is grounded in a range of human rights. SRHR is linked to national and international law and to achieving public health policy goals, such as the Sustainable Development Goals (SDGs) [36]. By addressing SRHR knowledge gaps, especially among young migrants, safer sexual behaviours and more timely access to prevention and health care services may be facilitated. There is little evidence of migrants’ knowledge of the legal right to abortion and other laws and policies concerning SRHR in the Swedish context. It is hypothesised that originating from countries with less restrictive abortion laws and having comprehensive sexual health education programmes, as well as having non-religious beliefs and a higher level of education, are associated with increased awareness about the legal right to abortion.

Our study aimed to assess knowledge of the right to safe and legal abortion and other key legal issues of SRHR among recently arrived migrants in Sweden. We also aimed to assess associations between incorrect knowledge of the Swedish abortion law and socio-demographic factors.

## Methods

### Study design and population

We conducted a cross-sectional survey on SRHR tailored to migrants in Sweden. In this study, we focused on migrants, defined by the International Organization for Migration (IOM) as “a person who moves away from his or her place of usual residence, whether within a country or across an international border, temporarily or permanently, and for a variety of reasons” [37]. The study population and eligible participants were aged 15 and older, born outside of Sweden, and who had migrated to Sweden.

### Survey instrument

The survey contained 60 questions on self-reported health needs, sexual risk-taking behaviours and attitudes towards norms and values, knowledge of HIV and five questions about knowledge of SRHR-related laws. The questionnaire was created in Swedish and English and then translated into Arabic, Dari, Somali, Spanish, and Tigrinya, given that these were the main languages in the study populations. A pilot study was conducted with participants from each of the translated languages in a Swedish language school for foreigners, *Svenska för invandrare* (SFI), to check for potential language misinterpretations and cultural misunderstandings. After performing language and cultural adaption, the surveys were back-translated and cross-checked with the English version to ensure accurate meanings. The survey answers were also reviewed during the pilot testing, allowing us to identify problematic questions to refine the survey.

#### Questions on SRHR-related laws

**Fig. 1:**
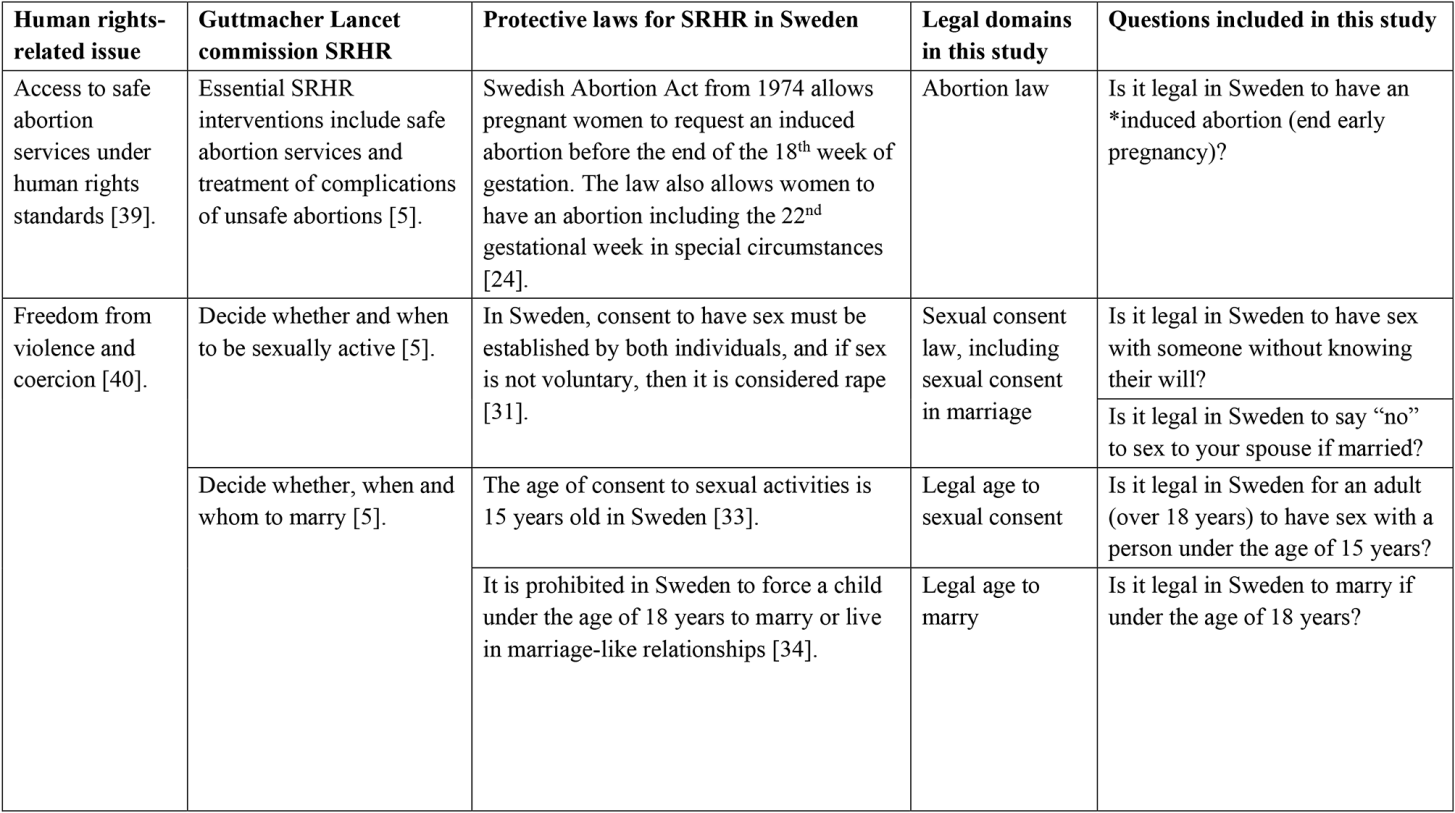
Definitions and study questions about knowledge of laws related to SRHR. (*Induced abortion is defined as the intentional termination of pregnancy [41]. Abbreviations: Sexual and Reproductive Health and Rights (SRHR))

We used the knowledge-attitude-practices model [38] to formulate the questions of the SRHR-related laws, which allowed us to collect information on what is known about the legal aspects of SRHR among migrants in Sweden. In addition, we applied the definitions from the Guttmacher Institute on SRHR and United Nations Human Rights as our framework for the legal questions [5, 35]. We assessed knowledge of the laws and policies concerning SRHR focused on the following domains: (1) Abortion law (2) Sexual consent law, (3) Sexual consent in marriage, (4) Legal age to sexual consent, and (5) Legal age to marry (Fig. 1).

### Data collection

The data was collected from December 2018 to November 2019. We targeted recently arrived migrants who attended SFI, or Swedish as a second language, *Svenska som andra språk* (SVA), or high schools across Sweden. Purposeful sampling was used to select the 31 diverse municipalities across Sweden. The SFI, SVA and high schools were randomly selected, and migrants were invited to participate in the study. The survey was programmed into a secured website and was accessible by digital tools, such as a computer, mobile or iPad. The website link to the survey was provided to the participants, and a research team member was also available on-site to provide clarifications and additional information on informed consent.

### Study variables

#### Dependent variable

The dependent variable was the incorrect response on whether abortion is legal in Sweden. We formulated the question “Is it legal in Sweden” as broad and unpersuasive legal statements that avoided complicated terminology or subject interpretation. This allowed us to have a simple phrase for asking legal questions and for the individual to select either ‘yes’, ‘no’ or ‘do not know’. We then coded the responses to ‘yes’ as correct, and ‘no’ or ‘do not know’ were considered incorrect.

#### Independent variables

Age, sex, educational level, comprehensive sexual health education before migrating to Sweden and religion were included in the analyses. In addition, country origin by how restrictive laws of abortion, duration in Sweden, reasons for migration, and residence permit status were included. We also included living situations of living with friends or roommates, with partners, relatives and family members, and multi-residence dwellings for asylum seekers such as the homes for care or residence known as *HVB (Hem för vård eller boende)* in Sweden [42].

Age (median age of 33 years) was divided into categories to compare age groups: 15 to 22; 23 to 32; 33 to 42; ages 43 and above. Education level was coded as compulsory education (≤ 9 years of formal school education) or no schooling as a low level of education, and post-secondary education (> 12 years) or secondary education (10 to 12 years) as a high level of education. Comprehensive sexual health education was dichotomised as previous and no previous education related to sexual and reproductive health. Islam, Christianity and other religions (i.e. Buddhism, Hinduism, Judaism or others) were considered religious versus non-religious or atheist. Native origin was grouped as the country region where the person was born and/or raised, and these categories were based on the United Nations geographical regions as the Middle East and North Africa (MENA), Sub-Saharan Africa (SSA), South Asia, and other country regions (Europe, Latin America and the Caribbean) [43]. We also grouped countries into different categories based on assessing how restrictive abortion laws were before 2018 and as compiled by the Guttmacher Institute Restrictive Abortion Laws [44]. We considered predominantly restrictive laws in countries where abortion is prohibited altogether or permitted to save a woman’s life or preserve physical health. We considered countries with less restrictive abortion laws where abortion is allowed on mental health and broad socio-economic grounds or had no restrictions as to the reason (see Supplementary Tables 1, Additional file 1). We then dichotomised country origin as countries with less restrictive abortion and predominantly restrictive laws [44]. We categorised the duration of living in Sweden as a short duration of less than one year and a long period of 2 or more years, and this was based on the year arrived in Sweden. The reason for migration was defined into the categories of seeking asylum versus non-asylum, such as family reunion, work, and other causes of migration. Resident status in Sweden was coded as no permit and having either a temporary (usually a limited period of 2 years and up to 3 years if given a refugee status) or permanent (continuous for five years) residence in Sweden [45]. European citizens and Swedish naturalised (acquired citizenship by application) were considered to have permanent residence status. We coded living situations of either living alone or not alone.

### Data analysis

Descriptive statistics were used to summarise the characteristics of the correct responses to the right to safe and legal abortion and other factors associated with it, including sexual consent, sexual consent in marriage, the legal age to sexual consent, and the age to marry. The results of the dependent variable (incorrect response on whether abortion is legal in Sweden) were presented as weighted frequencies and percentages. Univariable and multivariable adjusted odds ratios (AOR) with 95% confidence intervals (CI) for several potential predictors (age, sex, education level, sexual health education, religion, country origin by restrictions on abortion, duration of living in Sweden, reason for migration, residence permit in Sweden, and living situation) in relation to the probability of incorrect knowledge of abortion law were estimated with logistic regression models. Test of hypothesis at 5% level for the log adjusted odds ratios equal to zero were conducted with a two-sided Wald-type test with reference to a standard normal distribution. Additional multivariable logistic regression models estimated the associations between incorrect knowledge of sexual consent in marriage and socio-demographic characteristics among recent migrants and not knowing other key legal aspects (sexual consent law, the legal age for sexual consent and marriage), and these are presented in the supplementary material.

#### Ethics

The study protocol, including recruitment and consent procedures, was approved by the Swedish Ethical Review Authority (Dnr: 2017/2030-31). Detailed information about the study was provided to the respondents, including where to get support for SRHR services and accurate information about the Swedish laws related to SRHR. Written informed consent was collected. Participation was voluntary, and the respondents could withdraw from the study at any time without consequences; their responses would be treated as confidential, and the results would be reported anonymously.

## Results

### Socio-demographic characteristics

Of the 6,263 participants in the survey, 3557 responded to the question on knowledge of the Swedish abortion law (see Supplementary Fig. 1, Additional file 2). Most of the respondents had received formal education, with nearly 32% attending compulsory education (≤ 9 years of formal school), 31% secondary education (10 to 12 years), and 31% post-secondary education (> 12 years) (Table 1). About 46% of the respondents reported having received sexual health education before Sweden, while 54% did not have previous education about sexual health or did not know. Islam was the most common religion (60%), followed by Christianity (18%). Most (46%) of the respondents originally came from the MENA, while about 16% were from SSA, and 11% were from South Asia. Other country regions (26%) included Europe, Latin America and the Caribbean. About 74% of the respondents came from a country with predominantly restrictive abortion laws, and more than half (55%) came to Sweden between 2016 and 2019. Nearly half (47%) of the respondents were asylum seekers; overall, 60% had a permanent residence permit at the time of the survey. About 14% of the respondents lived alone, while most (77%) lived with family members.

**Table 1:** Socio-demographic characteristics of the respondents and knowledge of abortion law in Sweden, 2018

Characteristics	Missing	N	Knowledge of Swedish abortion law		Total
			Correct	Incorrect	
**Total**			925 (26%)	2632 (74%)	3557 (100%)
**Age (y)**	2706	3557			
15–22			219 (26%)	621 (74%)	840 (100%)
23–32			213 (28%)	552 (72%)	765 (100%)
33–42			217 (27%)	591 (73%)	808 (100%)
43+			276 (24%)	868 (76%)	1144 (100%)
**Sex**	2754	3509			
Women			556 (28%)	1453 (72%)	2009 (100%)
Men			359 (24%)	1141 (76%)	1500 (100%)
**Years of education completed**	2912	3351			
No school			57 (25%)	173 (75%)	230 (100%)
≤ 9 years of formal school			235 (22%)	825 (78%)	1060 (100%)
10 to 12 years			266 (26%)	757 (74%)	1023 (100%)
> 12 years			326 (31%)	712 (69%)	1038 (100%)
**Sexual health education**	2729	3534			
Previous sexual health education			521 (32%)	1097 (68%)	1618 (100%)
No previous sexual health education or don’t know			401 (21%)	1515 (79%)	1916 (100%)
**Religion**	3401	2862			
Christianity			168 (32%)	351 (68%)	519 (100%)
Islam			376 (22%)	1346 (78%)	1722 (100%)
Other religions (Buddhism, Hinduism, Judaism)			137 (28%)	355 (72%)	492 (100%)
Non-religious or atheist			52 (40%)	77 (60%)	129 (100%)
**Native origin**	2862	3401			
MENA			349 (22%)	1213 (78%)	1562 (100%)
South Asia			83 (22%)	299 (78%)	382 (100%)
SSA			151 (27%)	406 (73%)	557 (100%)
Other country regions			303 (34%)	597 (66%)	900 (100%)
Country origin by restrictions on abortion*	2862	3401			
Less restrictive abortion laws			295 (34%)	583 (66%)	878 (100%)
Predominantly restrictive abortion laws			591 (23%)	1932 (77%)	2523 (100%)
**Year arrived in Sweden**	2716	3547			
2016–2019			507 (26%)	1452 (74%)	1959 (100%)
2013–2015			340 (26%)	961 (74%)	1301 (100%)
2012 or earlier			76 (26%)	211 (74%)	287 (100%)
**Reason for migration**	3213	3050			
To seek asylum			363 (25%)	1084 (75%)	1447 (100%)
Family ties			219 (28%)	562 (72%)	781 (100%)
Work or study			96 (27%)	254 (73%)	350 (100%)
Other reasons for migration			115 (24%)	357 (76%)	472 (100%)
**Resident status in Sweden**	3038	3225			
No residence permit			50 (27%)	133 (73%)	183 (100%)
Temporary residence permit			258 (23%)	841 (77%)	1099 (100%)
Permanent residence permit			550 (28%)	1393 (72%)	1943 (100%)
**Living situation**	3075	3188			
With friends or roommates			55 (32%)	115 (68%)	170 (100%)
With partner, relatives, and/or family members			657 (27%)	1793 (73%)	2450 (100%)
Other (multi-residence dwelling)			33 (26%)	93 (74%)	126 (100%)
Alone			105 (24%)	337 (76%)	442 (100%)

*N = 6263. Data not available for all respondents due to missing values. Country origin by restrictions on abortion [44]. Correct answers on the knowledge of the abortion law refer to: It is legal in Sweden to have an induced abortion (end early pregnancy)? Yes (correct), No/Do not know (incorrect)

Abbreviations: Middle East and North Africa (MENA), Sub-Saharan Africa (SSA)

### Knowledge of key legal issues of SRHR

**Fig. 2:**
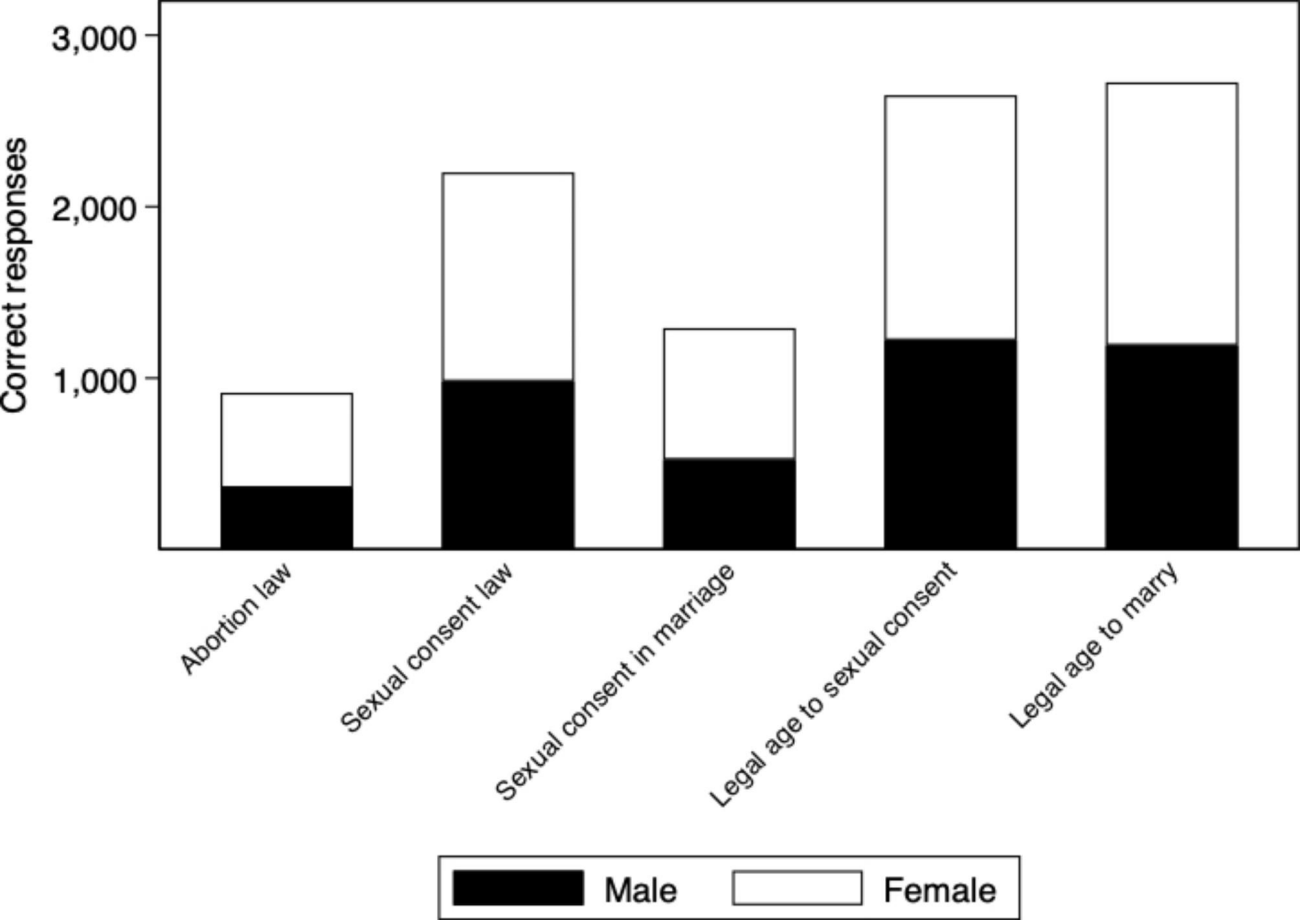
Participants’ correct responses to the laws and policies concerning SRHR

Of the 3557 respondents, 26% correctly responded to the legal status of induced abortion in Sweden. About 61% correctly answered the law regarding sexual consent, i.e. it is illegal to have sex without consent. However, nearly half (48%) did not know that a married person also has the right to decline to have sex with their spouse. Most, 89% of the respondents, knew that it is illegal to have sex with a minor (under the age of 15 years), and 88% were aware of the legal age to marry a minor, i.e. that it is illegal to marry someone under the age of 18 (Fig. 2).

#### Knowledge of abortion law

Based on the multivariable logistic regression model (Table 2), several factors were associated with a higher probability of incorrect knowledge of abortion law among recent migrants in Sweden: being religious (AOR, 2.12; 95% CI, 1.42–3.15) versus non-religious or atheist; not having previous sexual health education (AOR, 1.66; 95% CI, 1.36–2.04); and coming from a country where abortion is predominantly restrictive (AOR, 1.46; 95% CI, 1.16–1.85). In addition, low educational level (AOR, 1.29; 95% CI, 1.04–1.61) and having a temporary residence permit (AOR, 1.27; 95% CI, 1.02–1.58) versus a permanent residence permit were both associated with not knowing that it is legal to have an induced abortion in Sweden.

**Table 2:** Associations between socio-demographic characteristics and not knowing the abortion law among migrants in Sweden, 2018

	Univariable		Multivariable	
Variable	OR	95% CI	AOR	95% CI
**Age group, years**				
15–22	Ref			
23–32	0.91	0.73–1.13	0.99	0.75–1.32
33–42	0.96	0.77–1.19	1.16	0.87–1.55
43+	1.10	0.90–1.36	1.18	0.88–1.58
**Sex**				
Women	Ref			
Men	1.21	1.04–1.41	1.21	0.98–1.50
**Educational level**				
High level (> 10 years)	Ref			
Low level (no school or ≤ 9 years)	1.37	1.17–1.61	1.29	1.04–1.61*
**Sexual health education before Sweden**				
Previous sexual health education	Ref			
No previous sexual health education	1.79	1.54–2.08	1.68	1.38–2.05*
**Religion**				
Non-religious or atheist	Ref			
Religious	2.03	1.41–2.92	2.12	1.42–3.15*
**Country origin by restrictions on abortion**				
Less restrictive abortion laws	Ref			
Predominantly restrictive abortion laws	1.65	1.39–1.95	1.46	1.16–1.85*
**Duration living in Sweden**				
≥ 2 years	Ref			
< 1 year	1.03	0.87–1.21	1.09	0.85–1.39
**Reason for migration**				
Not asylum seeker	Ref			
Asylum seeker	1.09	0.93–1.28	0.87	0.69–1.10
**Resident status in Sweden**				
Permanent residence permit	Ref			
Temporary residence permit	1.28	1.08–1.51	1.27	1.02–1.58*
No residence permit	0.96	0.68–1.34	0.99	0.61–1.62
**Living situation**				
With friends, roommates, family or multi-residence	Ref			
Alone	1.19	0.95–1.51	1.19	0.88–1.60

*P-value significant at < 0.05

Abbreviations: OR Crude odds ratio, CI confidence interval, Ref reference, OR Odds ratio, AOR Adjusted odds ratio

**Fig. 3:**
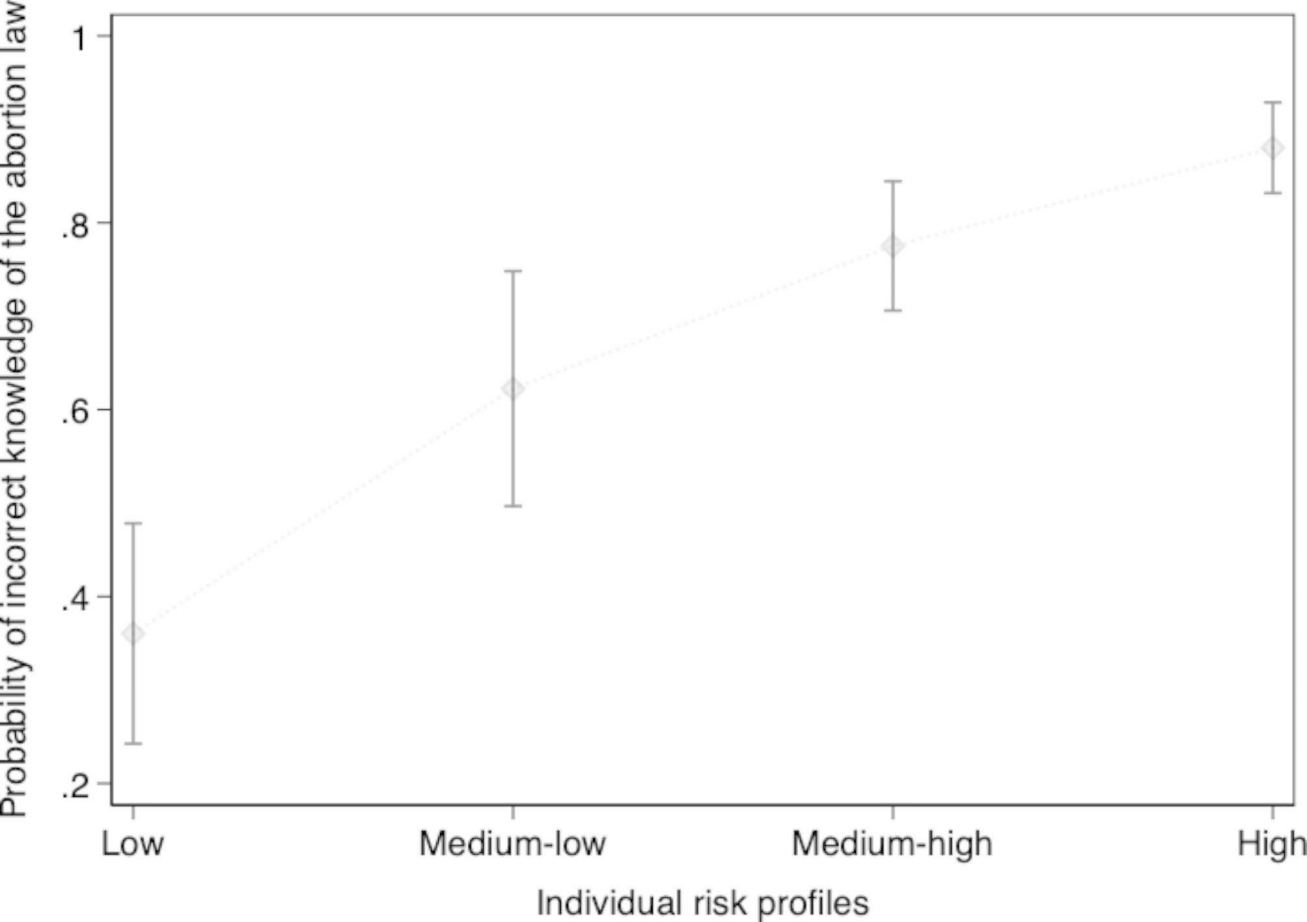
Predicted probability and confidence intervals of incorrect knowledge of the abortion law among migrants according to individual risk profiles. The vertical bars indicate the 95% confidence interval - Low: Medium-low: age 43 + years, low educational level (no school or ≤ 9 years), no previous sexual health education. Medium-high: age 43 + years, low educational level (no school or ≤ 9 years), no previous sexual health education, and religious. High: age 43 + years, low educational level (no school or ≤ 9 years), no previous sexual health education, religious, country origin from predominantly restrictive abortion laws, < 1-year duration in Sweden, asylum seeker, temporary residence permit and living alone

Age was not strongly associated with incorrect knowledge of abortion law compared to age groups between 15 and 22 years. Not having a residence permit (AOR, 0.99; 95% CI, 0.61–1.62) versus a permanent residence permit was not associated with the outcome variable. Similarly, we found no association with the outcomes by sex (male) (AOR, 1.21; 95% CI, 0.98–1.50), having a short duration (less than one year) living in Sweden (AOR, 1.09; 95% CI, 0.85–1.39), being an asylum seeker (AOR, 0.87; 95% CI, 0.69–1.10), and living alone (AOR, 1.19; 95% CI, 0.88–1.60) were compatible with no association. The probability (95% CI) was used to calculate individual risk profiles of incorrect knowledge of the abortion law using the predictor variables from the statistical analysis (Fig. 3). The additional multivariable logistic regression models indicated that incorrect knowledge of sexual consent in marriage was significantly associated with being religious (AOR, 3.12; 95% CI, 1.84–5.27), having no previous sexual health education (AOR, 1.82; 95% CI, 1.47–2.25), being an asylum seeker (AOR, 1.39; 95% CI, 1.09–1.77), living alone (AOR, 1.36; 95% CI, 1.00-1.85), and low level of education (AOR, 1.32; 95% CI, 1.05–1.67) (see Supplementary Tables 2, Additional file 3). In addition, not knowing other key legal aspects (sexual consent law, the legal age for sexual consent and marriage) was significantly associated with no previous sexual health education, coming from a country with predominantly restrictive abortion laws, having a low level of education, having a temporary residence permit and < 1-year duration in Sweden (see Supplementary Tables 3, Additional file 4).

## Discussion

Abortion rights are at the centre of the international SRHR agenda [46, 47]. We found that nearly three-quarters (74%) of recent migrants did not know that abortion is legal in Sweden, and close to half (48%) did not know that a married person could decline sex with their spouse. We also found that a higher probability of having incorrect knowledge about the Swedish abortion law was associated with the following factors: being religious; not having previous sexual health education; coming from a country with predominantly restrictive abortion laws; having a low educational level; and having a temporary residence permit. Age, sex, duration of living in Sweden, reasons for migration and living situation were not significantly associated with this incorrect knowledge. This study highlights the need for more targeted information in appropriate languages to migrant communities, especially migrants from non-European countries, about laws and policies related to SRHR.

Norms and values in relation to SRHR may explain the lack of knowledge of the abortion law and of sexual consent law, including the right to decline to have sex even within marriage and the legal age to sexual consent and marry. For example, it is illegal to marry before the age of 18 in many countries. However, traditions of child marriages are sometimes accepted in certain cultures and communities, despite there being prohibitions and minimum age requirements. Migrants in our study, both men and women, did not know a married person could decline to have sex with their spouse, and this was associated with a low level of education, not having sexual health education, being religious and asylum seeker, and living alone. Social norms, including patriarchal gender structures, sometimes underline the power dynamic and hierarchy over women’s ability to make decisions about their sexual relationships, sexual health, and reproductive health [48]. In line with our findings, prior studies have shown that migrant women’s sexual and reproductive health decision-making, including sexual activities within marriage and access to contraceptive methods, are often controlled by their husbands [49, 50]. Another explanation for incorrectly believing it is legal to have sex with one’s spouse against their will may suggest a lack of knowledge or obfuscation of a 2018 Swedish law on sexual consent. After implementing the new sexual consent law, young people had a different interpretation of sexual consent, such as consent being given through non-verbal cues, especially during casual sex encounters [32]. The new law may significantly impact verbal consent for sex, which may also have implications for language barriers. Therefore, it is important to identify new policies or laws that are poorly known and possibly misunderstood and reinforce accurate information at SFI, SVA, family planning services or clinics, and through comprehensive sexual health education or civic integration and educational programmes for migrants.

Respondents in this study who reported being religious were twice as likely not to know the legal right to abortion. The participants’ religious beliefs and perspectives on abortion may outweigh the possibility of becoming aware of the abortion law. Even though views on abortion differ, major religious traditions oppose abortion. In addition, within major religious traditions, there are frameworks and norms on the meaning of reproductive health, such as contraception, making religion an influencing factor towards SRHR [28]. For example, religious women reported negative attitudes towards family planning for limiting the number of children but not for child spacing [51]. Migrants who enter a new culture and environment may continue their own or previous set of constructs concerning sexual and reproductive health, which may also shape their knowledge, beliefs, and practices [52]. Over time, more societal exposure and gradual integration may eventually lead to enhanced awareness of laws concerning SRHR. Although time in Sweden was not a significant factor, this might be explained by the fact that our respondents had been living in Sweden for 2.5 years on average. Our findings also showed that the country of origin where abortion is more restricted influences migrants’ knowledge of the Swedish abortion law. Countries with legal restrictions on abortion may reinforce social norms and manifestations of abortion stigma, leading to the belief that abortion is morally wrong and legally restricted elsewhere.

Respondents who did not receive comprehensive sexual education before migrating to Sweden were less likely to know about the legal right to abortion. Even when comprehensive sexual education programmes appear to be readily available to young people, migrants may miss out on information about contraceptive methods, legal abortion, and safe abortion services as a result of religious and cultural norms and beliefs. This suggests that migrants often need comprehensive sexual health education and information about the SRHR-related laws and policies in Sweden, which may mitigate factors that place migrants, including young migrants, at risk of unwanted life changes such as sexual abuse or pregnancy. For this reason, sexual health education is a crucial component of improving SRHR outcomes [53], and critical for young people to gain knowledge and skills to make conscious, healthy and informed decisions about relationships and sexuality [54].

The data highlighted that migrants who had a temporary residence permit were more likely to have incorrect knowledge of the abortion law than those who had a permanent residence permit in Sweden, suggesting that there may be a considerable group of temporary migrants who are unfamiliar with the laws and policies concerning SRHR. Despite there being existing sexual and reproductive health services, migrants’ knowledge about these services is necessary to improve awareness of contraceptive methods and prevent unwanted pregnancies. Further research should also investigate efficient ways to raise migrants’ awareness of their rights, especially those with a temporary status, and empower them to utilise sexual and reproductive health services. Our results also showed that respondents in our study who had a lower level of education also had higher odds of incorrect knowledge of the Swedish abortion law and the sexual consent law, including consent within marriage. Low health literacy, which has been associated with lower educational levels, has also been suggested as contributing to low knowledge of SRHR among migrants in Sweden [55]. Low levels of education may influence migrants’ access to reliable health-related information, including rights and the legality of abortion. As education is a key determinant of health in later life [56], access to information and health services can be powerful tools, especially for informing people and raising awareness on abortion, laws and policies, and other SRHR.

In our analysis, the sex of the respondents was not associated with knowledge of the Swedish abortion law. Both men and women in our study may not be aware of the abortion law until there is an immediate need for abortion care services for women. Furthermore, after conducting risk profiles, our results indicated that the predicted probability is higher for migrants in our study to have incorrect knowledge of the abortion law, and this increased with more socio-demographic factors.

## Limitations

We assessed knowledge of the right to legal abortion, the current legal status of sexual consent and the legal age for both sex and marriage, but we did not include other legal aspects that govern SRHR, such as diagnosis and treatment of STIs, female genital mutilation, and other possible violations of SRHR. The way the questions were formulated in our study did not consider the safety of abortion and specific legal interpretations of abortion, such as gestational age limitations for induced abortion and abortion care. In addition, there is heterogeneity in the abortion laws for each country. For instance, we grouped countries into two categories based on the Guttmacher Institute definitions [44]. However, a country’s abortion laws undergo constant changes, and some countries allow abortion on the grounds of the woman’s physical health or mental health and foetal anomaly or impairments, which we collapsed into two categories. Furthermore, abortion and sexual consent are often sensitive and stigmatised topics, and this may have influenced the individuals’ perceptions and responses [57, 58]. Study limitations include that the survey was self-reported and prone to recall and selection bias. Given that we conducted our study in high school and Swedish language school settings, we were unable to collect information from participants with limited general literacy or undocumented migrants, and therefore, the results may not be generalisable. Our data may also underestimate migrants’ knowledge of the abortion law of those living long-term and permanently residing in Sweden.

## Conclusion

While abortion is legal in Sweden and safe services are available, only one-fourth of study respondents reported correct knowledge of safe and legal abortions, and half did not know that sexual consent is also required for married couples. Therefore, more efforts are needed for targeted and tailored interventions (e.g. comprehensive sexual health education and SRHR-related programmes), especially for recently arrived migrants with temporary status or originating from countries where abortion is heavily restricted. Migrants often need to give low priority to their SRHR in a new setting, and while the right to abortion continues to be threatened, access to safe abortion services is a human right. It is crucial for young people to have information and education about abortion law and other key legal issues related to SRHR (i.e. sexual consent law, legal age for sexual consent, legal age to marry), which can be achieved through various platforms. Sensitive and appropriate dissemination of the legal aspects of SRHR is also necessary across all religious traditions and educational levels. Further research is needed to evaluate associations of other laws and regulations on SRHR, including the information provided within comprehensive sexual health education.

## Electronic supplementary material

Below is the link to the electronic supplementary material.

Supplementary Material 1

Supplementary Material 2

Supplementary Material 3

Supplementary Material 4

## Data Availability

The datasets generated during this study are not publicly available due to the Swedish legal restrictions on personal data and current ethical approval for the study. Descriptive data in the format of plots, tables or graphs may be provided by the corresponding author upon justified request and following the procedures specified in the ethical approval.

## List of abbreviations

AOR: Adjusted odds ratio
CI: Confidence interval
GBV: Gender-based violence
HIV: Human immunodeficiency virus
HVB: Homes for care or residence *(Hem för vård eller boende)*
IOM: International Organization for Migration
MENA: Middle East and North Africa
OR: Odds ratio
RFSU: The Swedish Association for Sexuality Education *(Riksförbundet för sexuell upplysning)*
SFI: Swedish language school for foreigners *(Svenska för invandrare)*
SSA: Sub-Saharan Africa
SRHR: Sexual and reproductive health and rights
STI: Sexually transmitted infections
SVA: Swedish as a second language (*Svenska som andra språk)*
UN: United Nations
