# Supplementary material for: Knowledge of the abortion law and key legal issues of sexual and reproductive health and rights among recently arrived migrants in Sweden: a cross-sectional survey
Source: BMC Public Health. 2023 Mar 23;23:551. doi: 10.1186/s12889-023-15399-z (PMC10035217; doi:10.1186/s12889-023-15399-z)
Supplement: Supplementary file 1 — Supplementary Material 1 [file 12889_2023_15399_MOESM1_ESM.docx]

**S1 Table 1. Description of abortion laws by country**.

| **Description of abortion law by Guttmacher Institute** | **List of countries from Abortion Legality Worldwide*** | **Categories considered in this study** |
| --- | --- | --- |
| Prohibited altogether (no explicit legal exceptions (1) | Andorra, Angola, Congo-Brazzaville, Cong-Kinshasa, Dominican Republic, Egypt, El Salvador, Gabon, Guinea-Bissau, Honduras, Haiti, Iraq, Laos, Madagascar, Malta, Marshall Islands, Mauritania, Micronesia, Nicaragua, Palau, Philippines, Sao Tome and Principe, San Marino, Senegal, Suriname, Tonga | Predominantly restrictive abortion laws |
| To save life of woman (2) | Afghanistan, Antigua & Barbuda, Bangladesh, Bhutan, Brazil, Brunei Darussalam, Chile, Côte d’Ivoire, Dominica, Guatemala, Indonesia, Iran, Ireland, Kiribati, Lebanon, Libya, Malawi, Mali, Mexico, Myanmar, Nigeria, Oman, Panama, Papua New Guinea, Paraguay, Solomon Islands, Somalia, South Sudan, Sri Lanka, Sudan, Syria, Tanzania, Timor-Leste, Tuvalu, Uganda, United Arab Emirates, Venezuela, West Bank & Gaza, Yemen |  |
| To save life of woman/preserve physical health (3) | Argentina, Bahamas, Benin, Bolivia, Burkina Faso, Burundi, Cameroon, Central African Republic, Chad, Comoros, Costa Rica, Djibouti, Ecuador, Equatorial Guinea, Ethiopia, Grenada, Guinea, Jordan, Kenya, Kuwait, Lesotho, Liechtenstein, Maldives, Monaco, Morocco, Niger, Pakistan, Peru, Poland, Qatar, Rwanda, Saudi Arabia, South Korea, Togo, Vanuatu, Zimbabwe |  |
| To save life of woman/preserve physical and mental health (4) | Algeria, Botswana, Colombia, Eritrea, Gambia, Ghana, Israel, Liberia, Jamaica, Malaysia, Mauritius, Mozambique, Namibia, Nauru, New Zealand, Northern Ireland, Samoa, Seychelles, Sierra Leone, St. Kitts & Nevis, St. Lucia, Swaziland, Thailand, Trinidad & Tobago | Less restrictive abortion laws |
| To save life of woman/preserve physical and mental health/on socioeconomic grounds (5) | Barbados, Belize, Cyprus, Fiji, Finland, Great Britain, Hong Kong, Iceland, India, Japan, St. Vincent & Grenadines, Taiwan, Zambia |  |
| No restriction as to reason (with gestational and other requirements) (6) | Albania, Armenia, Australia, Austria, Azerbaijan, Bahrain, Belarus, Belgium, Bosnia-Herzegovina, Bulgaria, Cabo Verde, Cambodia, Canada, China, Croatia, Cuba, Czech Republic, Denmark, Estonia, France, Georgia, Germany, Greece, Guyana, Hungary, Italy, Kazakhstan, Kosovo, Kyrgyzstan, Latvia, Lithuania, Luxembourg, Macedonia, Moldova, Mongolia, Montenegro, Nepal, Netherlands, North Korea, Norway, Portugal, Puerto Rico, Romania, Russian Federation, Serbia, Singapore, Slovakia, Slovenia, South Africa, Spain, Sweden, Switzerland, Tajikistan, Tunisia, Turkey, Turkmenistan, Ukraine, United States, Uruguay, Uzbekistan, Vietnam |  |
| *Certain countries had legal grounds that were denoted by pregnancies resulting from rape, incest or when the fetus has a grave anomaly, and gestational-age limit [44]. | | |
