# Supplementary material for: Knowledge of the abortion law and key legal issues of sexual and reproductive health and rights among recently arrived migrants in Sweden: a cross-sectional survey
Source: BMC Public Health. 2023 Mar 23;23:551. doi: 10.1186/s12889-023-15399-z (PMC10035217; doi:10.1186/s12889-023-15399-z)
Supplement: Supplementary file 2 — Supplementary Material 2 [file 12889_2023_15399_MOESM2_ESM.docx]

**S2 Figure 1. Derivation of responses for analyses.**
