# Supplementary material for: Knowledge of the abortion law and key legal issues of sexual and reproductive health and rights among recently arrived migrants in Sweden: a cross-sectional survey
Source: BMC Public Health. 2023 Mar 23;23:551. doi: 10.1186/s12889-023-15399-z (PMC10035217; doi:10.1186/s12889-023-15399-z)
Supplement: Supplementary file 3 — Supplementary Material 3 [file 12889_2023_15399_MOESM3_ESM.docx]

| **S3 Table 2. Associations between socio-demographic characteristics and not knowing sexual consent in marriage among migrants in Sweden, 2018** | | | | |
| --- | --- | --- | --- | --- |
|  | **Univariable** | | **Multivariable** | |
| **Variable** | **OR** | **95% CI** | **AOR** | **95% CI** |
| **Age group, years** | | | | |
| 15-22 | Ref |  |  |  |
| 23-32 | 0.97 | 0.76–1.24 | 1.04 | 0.76–1.43 |
| 33-42 | 0.92 | 0.73–1.16 | 1.01 | 0.74–1.38 |
| 43+ | 0.97 | 0.78–1.20 | 1.28 | 0.94–1.75 |
| **Sex** |  |  |  |  |
| Women | Ref |  |  |  |
| Men | 1.24 | 1.06–1.46 | 1.09 | 0.87–1.36 |
| **Educational level** | | | | |
| High level (>10 years) | Ref |  |  |  |
| Low level (no school or ≤9 years) | 1.37 | 1.17–1.61 | 1.32 | 1.05–1.67* |
| **Sexual health education before Sweden** | | | | |
| Previous sexual health education | Ref |  |  |  |
| No previous sexual health education | 1.74 | 1.49–2.04 | 1.82 | 1.47–2.25* |
| **Religion** | | | | |
| Non-religious or atheist | Ref |  |  |  |
| Religious | 3.05 | 1.92–4.85 | 3.12 | 1.84–5.27* |
| **Country origin by restrictions on abortion** | | | | |
| Less restrictive abortion laws | Ref |  |  |  |
| Predominantly restrictive abortion laws | 1.18 | 0.99–1.41 | 0.92 | 0.72–1.18 |
| **Duration living in Sweden** | | | | |
| ≥2 years | Ref |  |  |  |
| <1 year | 0.86 | 0.72–1.02 | 1.17 | 0.89–1.53 |
| **Reason for migration** | | | | |
| Not asylum seeker | Ref |  |  |  |
| Asylum seeker | 1.38 | 1.16–1.64 | 1.39 | 1.09–1.77* |
| **Resident status in Sweden** | | | | |
| Permanent residence permit | Ref |  |  |  |
| Temporary residence permit | 1.02 | 0.85–1.21 | 1.03 | 0.82–1.30 |
| No residence permit | 0.95 | 0.66–1.35 | 0.71 | 0.40–1.29 |
| **Living situation** | | | | |
| With friends, roommates, family or multi-residence | Ref |  |  |  |
| Alone | 1.52 | 1.21–1.92 | 1.36 | 1.00–1.85* |
| **P-value* significant at <0.05.  Abbreviations: OR Crude odds ratio, CI confidence interval, Ref reference, OR Odds ratio, AOR Adjusted odds ratio. | | | | |
