# Supplementary material for: Knowledge of the abortion law and key legal issues of sexual and reproductive health and rights among recently arrived migrants in Sweden: a cross-sectional survey
Source: BMC Public Health. 2023 Mar 23;23:551. doi: 10.1186/s12889-023-15399-z (PMC10035217; doi:10.1186/s12889-023-15399-z)
Supplement: Supplementary file 4 — Supplementary Material 4 [file 12889_2023_15399_MOESM4_ESM.docx]

| **S4 Table 3. Associations between socio-demographic characteristics and not knowing laws related to SRHR among migrants in Sweden, 2018** | | | | | | | | | | | | |
| --- | --- | --- | --- | --- | --- | --- | --- | --- | --- | --- | --- | --- |
|  | **Sexual consent law** | | | | **Legal age for sexual consent** | | | | **Legal age to marry** | | | |
|  | **Univariable** | | **Multivariable** | | **Univariable** | | **Multivariable** | | **Univariable** | | **Multivariable** | |
| **Variable** | **OR** | **95% CI** | **AOR** | **95% CI** | **OR** | **95% CI** | **AOR** | **95% CI** | **OR** | **95% CI** | **AOR** | **95% CI** |
| **Age group, years** | | |  |  |  |  |  |  |  |  |  |  |
| 15-22 | Ref |  |  |  |  |  |  |  |  |  |  |  |
| 23-32 | 1.00 | 0.82–1.22 | 0.98 | 0.76–1.27 | 0.71 | 0.51–1.00 | 0.69 | 0.45–1.08 | 0.89 | 0.65–1.23 | 0.96 | 0.63–1.47 |
| 33-42 | 0.95 | 0.78–1.16 | 1.08 | 0.83–1.40 | 0.79 | 0.57–1.09 | 0.84 | 0.58–1.28 | 1.02 | 0.75–1.38 | 1.14 | 0.75–1.71 |
| 43+ | 0.88 | 0.73–1.05 | 1.01 | 0.78–1.30 | 0.57 | 0.41–0.78 | 0.75 | 0.50–1.15 | 0.68 | 0.50–0.92 | 0.72 | 0.46–1.13 |
| **Sex** |  |  |  |  |  |  |  |  |  |  |  |  |
| Women | Ref |  |  |  |  |  |  |  |  |  |  |  |
| Men | 0.87 | 0.76–1.00 | 0.80 | 0.66–0.96 | 0.91 | 0.72–1.15 | 0.85 | 0.62–1.17 | 0.91 | 0.72–1.13 | 0.95 | 0.69–1.31 |
| **Educational level** | | |  |  |  |  |  |  |  |  |  |  |
| High level (>10 years) | Ref |  |  |  |  |  |  |  |  |  |  |  |
| Low level (no school or ≤9 years) | 1.32 | 1.15–1.52 | 1.34 | 1.11–1.63* | 1.43 | 1.12–1.82 | 1.34 | 0.98–1.85 | 1.31 | 1.04–1.64 | 1.18 | 0.86–1.62 |
| **Sexual health education before Sweden** | | |  |  |  |  |  |  |  |  |  |  |
| Previous sexual health education | Ref |  |  |  |  |  |  |  |  |  |  |  |
| No previous sexual health education | 1.71 | 1.49–1.96 | 1.54 | 1.28–1.85* | 1.64 | 1.29–2.09 | 1.43 | 1.05–1.94* | 1.15 | 0.93–1.44 | 1.35 | 0.99–1.82 |
| **Religion** | | |  |  |  |  |  |  |  |  |  |  |
| Non-religious or atheist | Ref |  |  |  |  |  |  |  |  |  |  |  |
| Religious | 0.91 | 0.63–1.30 | 0.97 | 0.65–1.43 | 0.68 | 0.39–1.16 | 0.70 | 0.39–1.26 | 0.41 | 0.25–0.69 | 0.50 | 0.28–0.91* |
| **Country origin by restrictions on abortion** | | |  |  |  |  |  |  |  |  |  |  |
| Less restrictive abortion laws | Ref |  |  |  |  |  |  |  |  |  |  |  |
| Predominantly restrictive abortion laws | 1.27 | 1.08–1.49 | 1.38 | 1.10–1.72* | 1.03 | 0.78–1.34 | 1.13 | 0.78–1.64 | 0.55 | 0.43–0.69 | 0.52 | 0.37–0.72 |
| **Duration living in Sweden** | | |  |  |  |  |  |  |  |  |  |  |
| ≥2 years | Ref |  |  |  |  |  |  |  |  |  |  |  |
| <1 year | 1.18 | 1.02–1.37 | 1.51 | 1.22–1.88* | 0.84 | 0.64–1.10 | 0.78 | 0.54–1.14 | 0.94 | 0.73–1.21 | 0.75 | 0.52–1.08 |
| **Reason for migration** | | |  |  |  |  |  |  |  |  |  |  |
| Not asylum seeker | Ref |  |  |  |  |  |  |  |  |  |  |  |
| Asylum seeker | 0.90 | 0.78–1.04 | 0.89 | 0.72–1.09 | 0.75 | 0.58–0.96 | 0.71 | 0.50–1.00 | 0.70 | 0.55–0.90 | 0.65 | 0.46–0.91 |
| **Resident status in Sweden** | | |  |  |  |  |  |  |  |  |  |  |
| Permanent residence permit | Ref |  |  |  |  |  |  |  |  |  |  |  |
| Temporary residence permit | 1.20 | 1.04–1.39 | 1.26 | 1.04–1.53* | 0.71 | 0.54–0.92 | 0.82 | 0.59–1.15 | 0.76 | 0.59–0.98 | 0.72 | 0.51–1.01 |
| No residence permit | 0.96 | 0.71–1.29 | 1.39 | 0.90–2.14 | 1.12 | 0.69–1.81 | 0.93 | 0.46–1.89 | 1.62 | 1.05–2.50 | 1.03 | 0.53–2.02 |
| **Living situation** | | |  |  |  |  |  |  |  |  |  |  |
| With friends, roommates, family or multi-residence | Ref |  |  |  |  |  |  |  |  |  |  |  |
| Alone | 1.05 | 0.87–1.28 | 1.16 | 0.90–1.49 | 1.15 | 0.84–1.57 | 1.02 | 0.66–1.58 | 1.16 | 0.86–1.57 | 1.22 | 0.80–1.84 |
| **P-value* significant at <0.05.  Abbreviations: OR Crude odds ratio, CI confidence interval, Ref reference, OR Odds ratio, AOR Adjusted odds ratio. | | | | | | | | | | | | |
